# Phase contrast scanning transmission electron microscopy imaging of light and heavy atoms at the limit of contrast and resolution

**DOI:** 10.1038/s41598-018-20377-2

**Published:** 2018-02-08

**Authors:** Emrah Yücelen, Ivan Lazić, Eric G. T. Bosch

**Affiliations:** Thermo Fisher Scientific (formerly FEI), Achtseweg Noord 5, 5600KA Eindhoven, The Netherlands

## Abstract

Using state of the art scanning transmission electron microscopy (STEM) it is nowadays possible to directly image single atomic columns at sub-Å resolution. In standard (high angle) annular dark field STEM ((HA)ADF-STEM), however, light elements are usually invisible when imaged together with heavier elements in one image. Here we demonstrate the capability of the recently introduced Integrated Differential Phase Contrast STEM (iDPC-STEM) technique to image both light and heavy atoms in a thin sample at sub-Å resolution. We use the technique to resolve both the Gallium and Nitrogen dumbbells in a GaN crystal in [${\bf{10}}\bar{{\bf{1}}}{\bf{1}}$] orientation, which each have a separation of only 63 pm. Reaching this ultimate resolution even for light elements is possible due to the fact that iDPC-STEM is a direct phase imaging technique that allows fine-tuning the microscope while imaging. Apart from this qualitative imaging result, we also demonstrate a quantitative match of ratios of the measured intensities with theoretical predictions based on simulations.

## Introduction

Without any doubt (scanning) transmission electron microscopy ((S)TEM) is one of the most powerful structural characterization tools available to numerous science and engineering disciplines. Thanks to their negative charge, strong interaction occurs between the probing electrons and the electric field produced by the atoms, thereby making (S)TEM a tool that is uniquely suited for visualization of structures composed of different elements, from the very lightest to the very heaviest.

In the past 20 years the field of electron microscopy witnessed a drastic jump in the resolving power of transmission electron microscopes from about 200 pm to 50 pm^1^. The resolution limits imposed by the limitations of electromagnetic round lenses, have been overcome by successful construction and integration of aberration correction units into electron microscopes^2^. This increase in resolution resulted in increased measurement precision and development of new possibilities for analysis at the atomic scale. Nowadays, there are many examples of atomic scale imaging of nanostructures such as direct observation of the morphological changes of Pt nanoparticles surfaces during catalytic cycle^3^, the structure of dislocations in GaN^4^, structural anomalies such as octahedral tilting and strain in perovskites^5^ and piezoelectric polarization in semiconductor heterojunctions^6^, to name but a few.

Conventional (S)TEM imaging techniques provide atomic scale images of the phase of the transmission function, which can be interpreted as the projected potential for a thin sample, through different contrast transfer mechanisms. These contrast mechanisms are, in general, nonlinear (e.g. the contrast transfer depends on the sample itself, whereas a necessary (but not sufficient) condition for linear imaging is that it doesn’t) but in some special cases they are or can be made either approximately or completely linear.

One technique that is approximately linear is (high-angle) annular dark field, (HA)ADF-STEM. The observed contrast for this technique linearly images the square of the phase of the transmission function^7–10^. For thin samples this phase is directly proportional to the (projected) electrostatic potential. The result is an imaging technique that images roughly the square of the atomic number *Z*. This is why (HA)ADF-STEM is commonly referred to as Z-contrast imaging but it is at the same time also the reason why it is insensitive to light elements such as O, N, C, B and Li when imaged together with heavier atoms like Si, Ga, Sr, Au etc. The ultimate resolution of modern aberration corrected (S)TEM instruments can therefore often only be shown for elements of relatively high scattering power because of this insensitivity of (HA)ADF-STEM to light elements.

Apart from conventional techniques, also non-conventional STEM techniques were proposed to retrieve the phase from recording the whole CBED pattern or parts of it, such as e.g. Ptychography^11,12^ or its variants^13–16^. Although promising, numerically appealing and producing results, there are still challenges regarding these methods to be addressed. They involve sometimes iterative reconstruction schemes, which are not always converging^17^, are restricted to the weak phase object approximation^14,15^, and deal with ill-conditioned problems such as deconvolution^14–16^.

In this work we use a recently introduced novel scanning transmission electron microscopy technique called integrated differential phase contrast STEM (iDPC-STEM) imaging^18^, which is truly linear for thin samples (where a sample is considered thin if the result of a mono-slice simulation does not differ significantly from the multi-slice image of the sample, see also the supplementary material). The iDPC-STEM method is an extension of the standard DPC-STEM technique^19^ and enables direct imaging of the phase of the transmission function for non-magnetic samples. For thin samples, this yields an image that is directly interpretable as the (projected) electrostatic potential. Therefore, as direct imaging of this phase has always been one of the ultimate goals of electron microscopy^9,20–23^, iDPC-STEM comes close to achieving this goal. The resulting contrast is now roughly proportional to the atomic number Z, which drastically improves the detectability of light elements among heavy elements in one image. As an example, this enabled the precise mapping of the oxygen positions in NdGaO_3_-La_0.67_Sr_0.33_MnO_3_ which determine critical behavior of the material^24^.

An iDPC-STEM image, representing the *scalar* electrostatic potential field of the sample, is obtained by integration of the vector image acquired by DPC-STEM, (consisting of two components, viz. DPCx and DPCy), representing the *vector* electric field of the sample^8,18,25–27^. The acquisition of an iDPC-STEM image is, in its simplest form, enabled by a 4-quadrant detector. As we will see, this already fully enables live imaging of light and heavy elements together at the ultimate resolution. Moreover, using an HAADF detector, conventional ADF-STEM images can also still be recorded simultaneously and therefore both techniques together can be used to determine e.g. the unit cell contents of crystalline samples.

In the iDPC-STEM technique the signal to noise ratio (SNR) is superior compared to ADF-STEM imaging and also to other phase contrast high resolution TEM imaging as was recently demonstrated for imaging of dose-sensitive materials like zeolite and biological materials^8,28^. There are two main reasons for this. The first reason is that all available electrons are used during measurement of the center of mass (COM) of the convergent beam electron diffraction (CBED) pattern (using either a camera or a segmented detector), which is linear in the electric field at the position of the probe. The second, more important, reason is the physical regularization enforced by the integration process of the vector field. Only the conservative part of the vector field image (the part that can be written as the gradient of a scalar field) contributes to the iCOM- or iDPC-STEM image, while the non-conservative part of the field will be suppressed. The electrical field is conservative and survives the integration processes, whereas the noise has a (large) non-conservative part which will be removed.

In this work we demonstrate imaging at the limit of contrast and resolution on Wurtzite GaN samples prepared in two different orientations ([$10\bar{1}1$] and [$11\bar{2}0$]) using ADF-STEM and iDPC-STEM simultaneously. These orientations were chosen because they show the lighter Nitrogen atoms at non-symmetrical positions between the Gallium atoms, enabling us to confirm that we are looking at real atom columns and not at artifacts. With iDPC-STEM we will straightforwardly overcome the challenge of revealing light N next to heavy Ga columns in a single image, and demonstrate resolution of at least 63 pm for both elements. Being able to have both techniques live on the screen during acquisition also enables live focusing and fine-tuning of the probe, which is crucial to reach the limits of contrast and resolution.

## Results

### Acquisition and iDPC-STEM imaging

**Figure 1:**
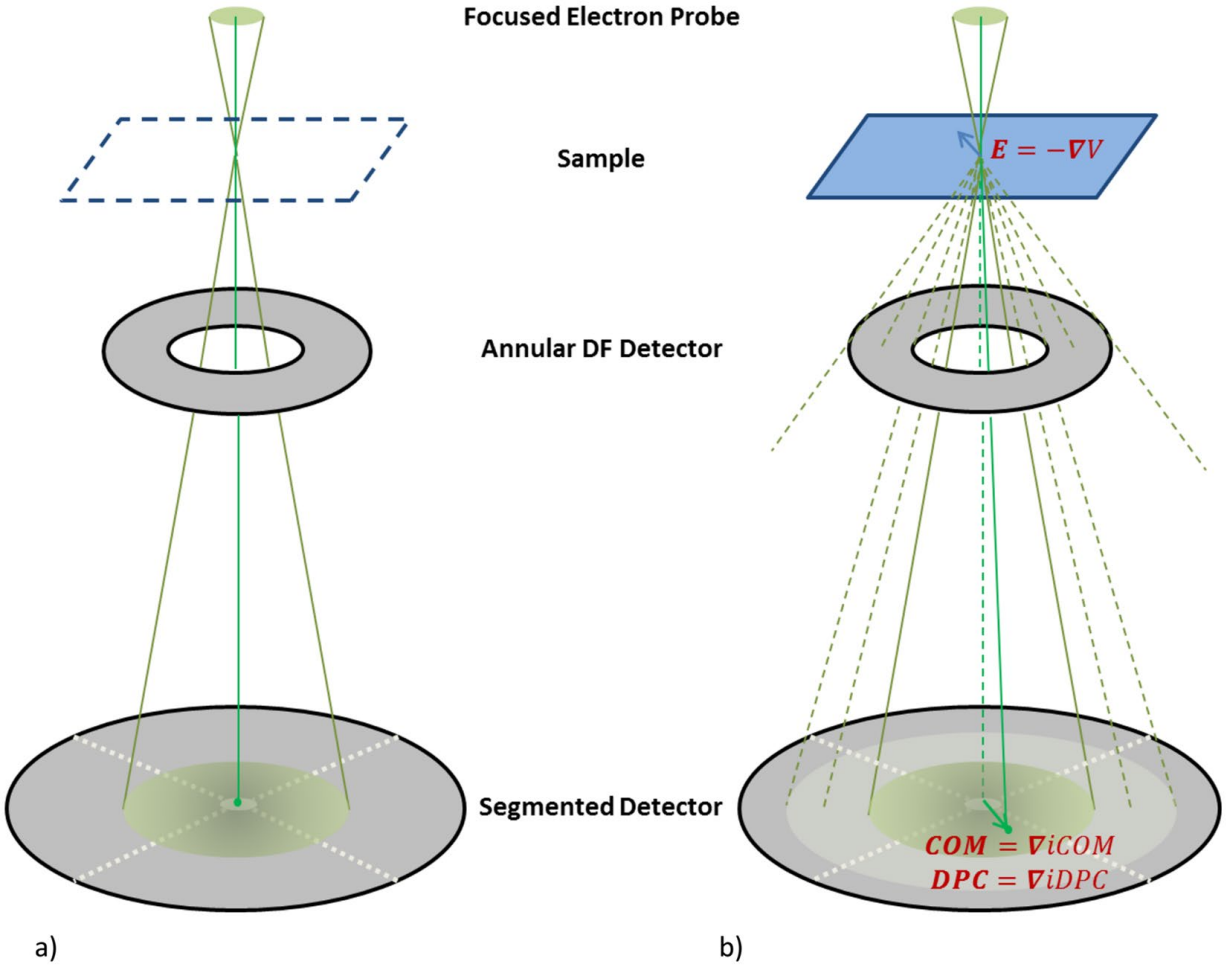
Schematic representation of the experimental setup. (**a**) Focused probe without sample. (**b**) Focused probe with a sample.

The experimental setup for simultaneous iDPC- and ADF-STEM imaging is presented in Fig. 1. An aberration corrected fine electron probe focused on the sample forms a CBED pattern in the far field, also sometimes called a Ronchigram. The far field plane is in practice brought to a finite distance using a lens projector system (not shown in the figure), effectively, to its back focal plane.

Without a sample, the CBED pattern is just an image of the beam limiting aperture. The illuminated far field area defined this way is called the bright field (BF) region, and is often referred to as the BF disk because the beam limiting aperture usually has a round shape. This situation is shown in Fig. 1a.

When a sample is present, as shown in Fig. 1b, electrons are scattered due to the presence of electromagnetic fields caused by the atoms in the sample. For non-magnetic samples the electrostatic potential field results in a phase shift of the electron wave passing through the sample. This phase shift, in turn, alters the intensity distribution in the CBED pattern and it was shown^29,30^ that there is a linear relation between the gradient of this phase shift and the position of the center of mass (COM) of the CBED pattern (indicated in the detector plane in Fig. 1b); this was also rigorously mathematically proven^8,18^.

For thin samples, the phase shift is directly proportional to the (projected) electrostatic potential in the sample. Therefore, its gradient is directly proportional to the (projected) electric field of the thin sample, and can be directly imaged by recording CBED patterns over the sample at probe positions ${\boldsymbol{r}}$ and computing COM vectors, forming a COM-STEM vector image ${{\boldsymbol{I}}}^{COM}({\boldsymbol{r}})$. This is because the electric field ${\boldsymbol{E}}({\boldsymbol{r}})$ is a conservative vector field, i.e.$\,{\rm{\nabla }}\times {\boldsymbol{E}}({\boldsymbol{r}})=0$, and therefore an electrostatic potential field $V({\boldsymbol{r}})$ exists such that ${\boldsymbol{E}}({\boldsymbol{r}})=-{\rm{\nabla }}V({\boldsymbol{r}})$. Similarly, a scalar integrated COM or iCOM-STEM image,$\,{I}^{iCOM}({\boldsymbol{r}})$, exists such that the COM-STEM vector image is its gradient: ${{\boldsymbol{I}}}^{COM}({\boldsymbol{r}})={\rm{\nabla }}{I}^{iCOM}({\boldsymbol{r}})$.

The resulting integrated image directly represents the electrostatic potential of the sample and the final connection between the iCOM image and the electrostatic potential of a thin sample is given in the Fourier domain by^8^

1 $$ {\mathcal F} \{{I}^{iCOM}({\boldsymbol{r}})\}({\boldsymbol{k}})=\frac{1}{2\pi }\overline{ {\mathcal F} \{{|{\psi }_{in}({\boldsymbol{r}})|}^{2}\}}({\boldsymbol{k}})\cdot  {\mathcal F} \{\sigma V({\boldsymbol{r}})\}({\boldsymbol{k}})$$

where ${\psi }_{in}({\boldsymbol{r}})$ is the electron input wave (the probe) at the sample, $\sigma =2\pi me\lambda /{h}^{2}$ is a constant containing the (relativistic) mass $m$, charge $e=|e|$, and the wavelength $\lambda$, of the electron and $h$ is Planck’s constant. Eq. (1) has the form required for ideal linear imaging (see Sec. 1.3 in ref.^8^). The first factor in (1) is the contrast transfer function (CTF), which is essentially the Fourier transform of the intensity of the probe in this case. The second factor contains the object, $\phi ({\boldsymbol{r}})=\sigma V({\boldsymbol{r}})$ which for thin samples is the phase of the transmission function,$\,T({\boldsymbol{r}})=\,{e}^{i\phi ({\boldsymbol{r}})}$. This is why iCOM-STEM can be considered as a direct phase imaging technique, which has always been the ultimate goal of electron microscopy^22^. Note that (1) is not valid at ***k*** = 0, corresponding to the fact that the electrostatic potential is also only defined up to an arbitrary constant and its reference level can be chosen freely.

The COM- and iCOM-STEM images are ideal versions of DPC- and iDPC-STEM^8,18^ images, respectively. Unlike the former, the latter are trying to capture the COM vector with only a few detector segments. The reason for applying this approach, described in more detail in the methods section and the supplementary material, is that pixelated detectors, although already used for this purpose^31^, currently are not yet up to speed for live imaging. Besides, if a complete CBED pattern is recorded at every position, the amount of data obtained becomes very large. It was shown^8,18^ that segmented STEM detectors, such as the 4-quadrant DF4 detector used for conventional DPC imaging shown in Fig. 1, provide a fast and, above all, accurate representation of the true COM movement.

### Acquisition and ADF-STEM imaging

As indicated in Fig. 1b a small fraction of the electrons are also scattered outside of the BF area, to the dark field (DF) region. These parts of the CBED pattern are caught by the high angle annular dark field (HAADF) detector in order to form an (HA)ADF-STEM image if required. For our measurements the camera length of the projection system was set to such a value that the bright-field disk, and a sufficient part of the DF area, passes through the hole of the HAADF detector and is detected by the segmented DF4 detector. The resulting range of collection angles for the HAADF detector is 50–200 mrad, making this effectively an ADF detector at this camera length.

Although the electrons that end up in DF should theoretically all be included in a precise detection of the COM, detecting part of the DF region on a separate annular dark field (HAADF) detector to enable simultaneous ADF- and iDPC-STEM imaging has a negligible effect on the COM measurement. This is the case as long as the inner angle caught by the (HA)ADF detector is sufficiently larger than the outer angle of the BF disk. This is the case with the inner angle of 50 mrad at the opening angle of 29.4 mrad that was used in our experiments.

### Simultaneous iDPC- and ADF-STEM imaging of [${\bf{10}}\bar{{\bf{1}}}{\bf{1}}$] and [${\bf{11}}\bar{{\bf{2}}}{\bf{0}}$] oriented GaN

To achieve the best available resolution in STEM mode, probe aberration correction is employed and aberrations are reduced such that the optimal usable convergence angle is 29.4 mrad. Together with the source size this results in a probe that is small enough to resolve a spatial resolution of at least 60 pm.

**Figure 2:**
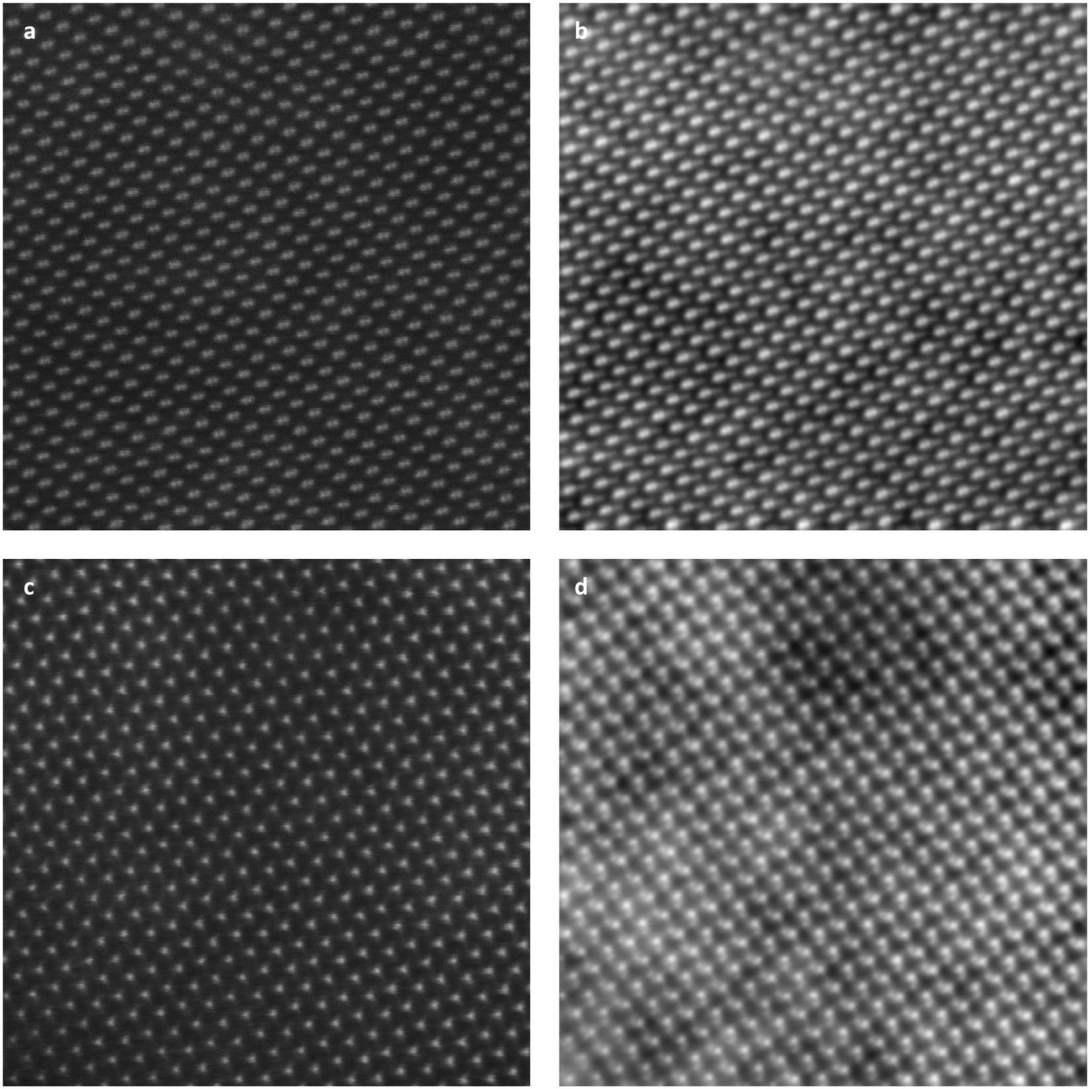
(**a**) ADF-STEM and (**b**) iDPC-STEM images of a GaN crystal in [$10\bar{1}1$] orientation. (**c**) ADF-STEM and (**d**) iDPC-STEM image of a GaN crystal in [$11\bar{2}0$] orientation. ADF- and iDPC-STEM images were recorded simultaneously in both cases. Beam current is 10 pA in all cases; the dwell time is 20 μs in (**a**,**b**), and 10 μs in (**c**,**d**); beam opening angle 29.4 mrad FOV is 6.2 nm. All images are shown as they appear live on the screen without any post-processing.

Figure 2 shows raw (i.e. unfiltered/not CTF corrected) iDPC- and ADF-STEM images for a Wurtzite GaN crystal in [$10\bar{1}1$] and [$11\bar{2}0$] projection as they appear on the screen during acquisition. Especially the [$10\bar{1}1$] projection demonstrates the resolution, as in this projection the Gallium atoms appear in pairs at a distance of 63 pm, which are resolved in all images.

The iDPC-STEM image in Fig. 2b, however, not only shows the Gallium but also the Nitrogen columns, which also appear in pairs at the same distance of 63 pm and 79 pm away from the Ga pairs. Up till now, only the separation between the two Ga atom columns has been directly shown using (HA)ADF-STEM imaging with an aberration corrected probe^32,33^. Due to the lower scattering power of N atoms, the two N columns have never directly been observed, and, hence, the separation between the two has also never been directly resolved in (HA)ADF-STEM. Instead, only a weak asymmetric shoulder due to the presence of the N atoms has been observed under extreme high SNR conditions achieved by employing, e.g. high beam currents or longer acquisition times. In Fig. 2b the two Nitrogen columns are everywhere recognizable (cf. also the line profile given in Supplementary fig. S3e).

The ADF- and iDPC-STEM images are recorded simultaneously and appear live on the screen. That fact enables proper focusing and precise fine tuning of the probe which is key to reach this high resolution. Even after the best fine tuning of the ADF-STEM conditions, the resulting ADF image contrast is not enough to reveal the N dumbbells, let alone resolve them. Seeing the N- next to the Ga-dumbbells in iDPC-STEM provides further focusing and fine tuning possibilities which resulted in the images presented in Fig. 2.

The difference in visibility of the N atomic columns between the two techniques can be qualitatively understood (and will be analyzed quantitatively later in the text). In ADF-STEM, the square of the electrostatic potential is imaged^7,18^ (see the methods section). Hence, the resulting signal is roughly proportional to the square of the atomic number, Z, and the resulting contrast is therefore roughly (31/7)^2^ ≈ 20. For iDPC-STEM, on the other hand, the signal is roughly linear in Z and the contrast is (31/7) ≈ 4.5. This means that a higher dynamic range is needed in ADF-STEM to reveal the N columns together with the Ga columns. The dynamic range of the detector is large enough to accommodate the factor 20 but achieving the necessary SNR is challenging given the weak DF signal.

As the DF signal is orders of magnitude weaker than the BF signal, the ADF-STEM image is much more prone to shot noise than the iDPC-STEM image. The shot noise cannot easily be reduced without compromising the ultimate resolution (e.g. by using a higher beam current or longer dwell times). The resulting SNR is not large enough to show both Ga and N at the same time with ADF-STEM. The iDPC-STEM signal has a much better SNR, which together with the much lower contrast between Ga and N enables imaging of both species at the same time.

The 3 pm pixel size and 2048 × 2048 scan grid result in a field of view (FOV) of 6.2 nm, which is large enough to be able to directly study e.g. lattice defects and imperfections (not present in these images). It would be difficult to reach a similar FOV with comparable pixel sizes such as presented in this work, with a ptychographic reconstruction and have it shown live on the screen as the amount of data that needs to be recorded is a few orders of magnitude higher than what is required for iDPC-STEM imaging, and an off-line reconstruction needs to be computed. Recently a ptychographic reconstruction of GaN in [$11\bar{2}0$] orientation was presented by Yang *et al*.^13^ showing both Gallium and Nitrogen columns. Their reconstruction, however, shows Ga columns that are at some places weaker than the N columns and with inverted contrast at some places. In the iDPC-STEM images of Fig. 2b,d the Ga columns are everywhere brighter than the N columns next to them, demonstrating that the resulting image is free of artifacts and directly interpretable.

Another benefit of direct linear imaging of the electrostatic potential is its ability to reveal low frequency information caused by e.g. sample thickness variations, build-in potentials, contamination, strain at interfaces, surface modification related to sample preparation, beam damage, etc. Therefore, the low frequency signal provides useful information, although sometimes it can be undesired or overwhelming depending on the application. In our case in Fig. 2 raw images are shown, containing complete information about the sample. In the supplementary material we also show the effect of high-pass filtering, which is the standard remedy whenever low pass information is irrelevant or undesired in (S)TEM.

As the contrast transfer of both ADF- and iDPC-STEM is positive definite when in focus, and well understood^7,8,18^, it is also possible to correct for the contrast transfer function (CTF). Both techniques have a CTF that is proportional to the Fourier transform of the intensity of the probe, which is discussed in more detail in the methods section. For iDPC-STEM it is given by (1) or to be precise by (3) in the methods; for ADF-STEM the difference in the CTF is the proportionality constant, Eq. (4) in the methods. This constant is still slightly sample-dependent (see supplementary material) and hence makes ADF-STEM a weakly nonlinear technique. Note that the fact that the CTF is basically the Fourier transform of the intensity of the probe follows from the same theory as for the other techniques, which assumes a fully coherent source and does not include inelastic effects. Nevertheless, the inelastically scattered electrons (e.g. by inner-shell ionizations) still contribute to the signal in ADF-STEM and are also needed in order to reproduce the measured SNR. There is no such contribution for iDPC-STEM, as the inelastically scattered electrons are not able to shift the COM as the majority of inelastic processes have no preferred azimuthal direction.

CTF correction (i.e. deconvolution) is in principle achieved by dividing the Fourier transform of the image with the known CTF and then transforming back. Unlike in TEM or BF-STEM, the only region where the CTF is zero in iDPC- and ADF-STEM is the region beyond the spatial frequency corresponding to two times the opening semi-angle of the beam. That region, therefore, contains noise only and can be safely filtered out. The regions where the CTF is close to zero should also be avoided as dividing by these small values will result in blowing up the noise at high spatial frequencies (a well-known problem with deconvolution in general). A low pass filter parameter is therefore defined to keep us within a safe distance from the region where the CTF becomes close to zero, together with a parameter defining up to which frequency we would like to correct, and a flat plateau in between. Full details of the applied CTF correction are explained in more detail in the supplementary material.

**Figure 3:**
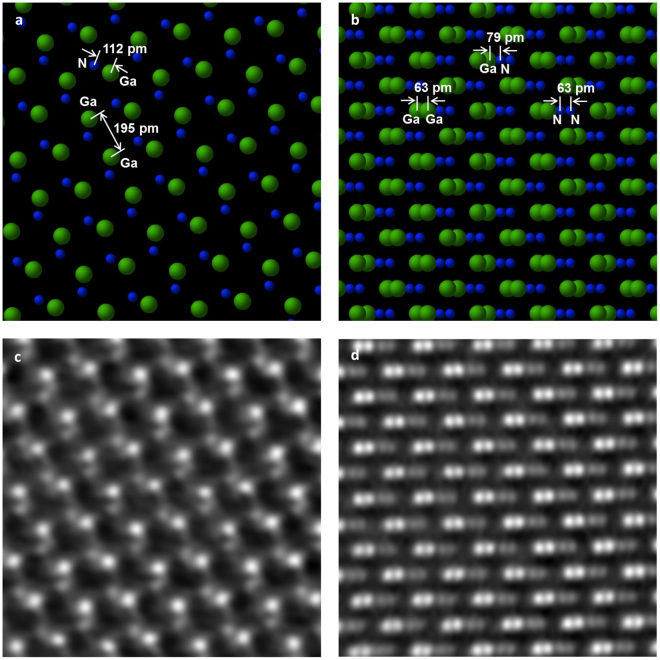
(**a**) Schematic representation of GaN crystal in [$11\bar{2}0$] direction. (**b**) Schematic representation of GaN crystal in [$10\bar{1}1$] direction. (**c**) iDPC-STEM image (CTF-corrected and high pass filtered) of GaN [$11\bar{2}0$] and (**d**) iDPC-STEM image (CTF-corrected and high pass filtered) of [$10\bar{1}1$] GaN. FOV is 2.0 nm. In (**c**) some features are visible that can be attributed to the fact that this sample for the [$11\bar{2}0$] orientation was slightly thicker than the other orientation. Based on simulations we estimate the thickness to be around 15 nm whereas it is around 7 nm for the[$10\bar{1}1$] orientation.

Figure 3 shows the lattice structure of GaN in both orientations together with a matching cut-out from corresponding iDPC images taken at a higher magnification than Fig. 2, and corrected for the CTF. Furthermore, as we mentioned above, some high-pass filtering is applied, which reduces the background low-frequency information which is due to sample thickness variations and topological changes due to build-up of contamination and surface modification related to sample preparation. Comparison of images with CTF correction and images that are just low-pass filtered (shown in the supplementary material in Figs S3 and S4, respectively) shows that the effect of CTF correction is dominant in making the nitrogen dumbbells become more pronounced and well resolved.

The resulting images clearly show that iDPC-STEM is able to resolve the complete structure of the GaN crystal in both orientations, including the positions of the nitrogen columns. Figure 3 proves directly and in real space that the microscope reaches a resolution of at least 63 pm both for light and heavy elements as they are imaged together within one iDPC-STEM image. At the same time it proves that, even at this very challenging edge of contrast and resolution, the limitations of ADF-STEM imaging (i.e. the object, and low SNR) are fully overcome by iDPC-STEM.

**Figure 4:**
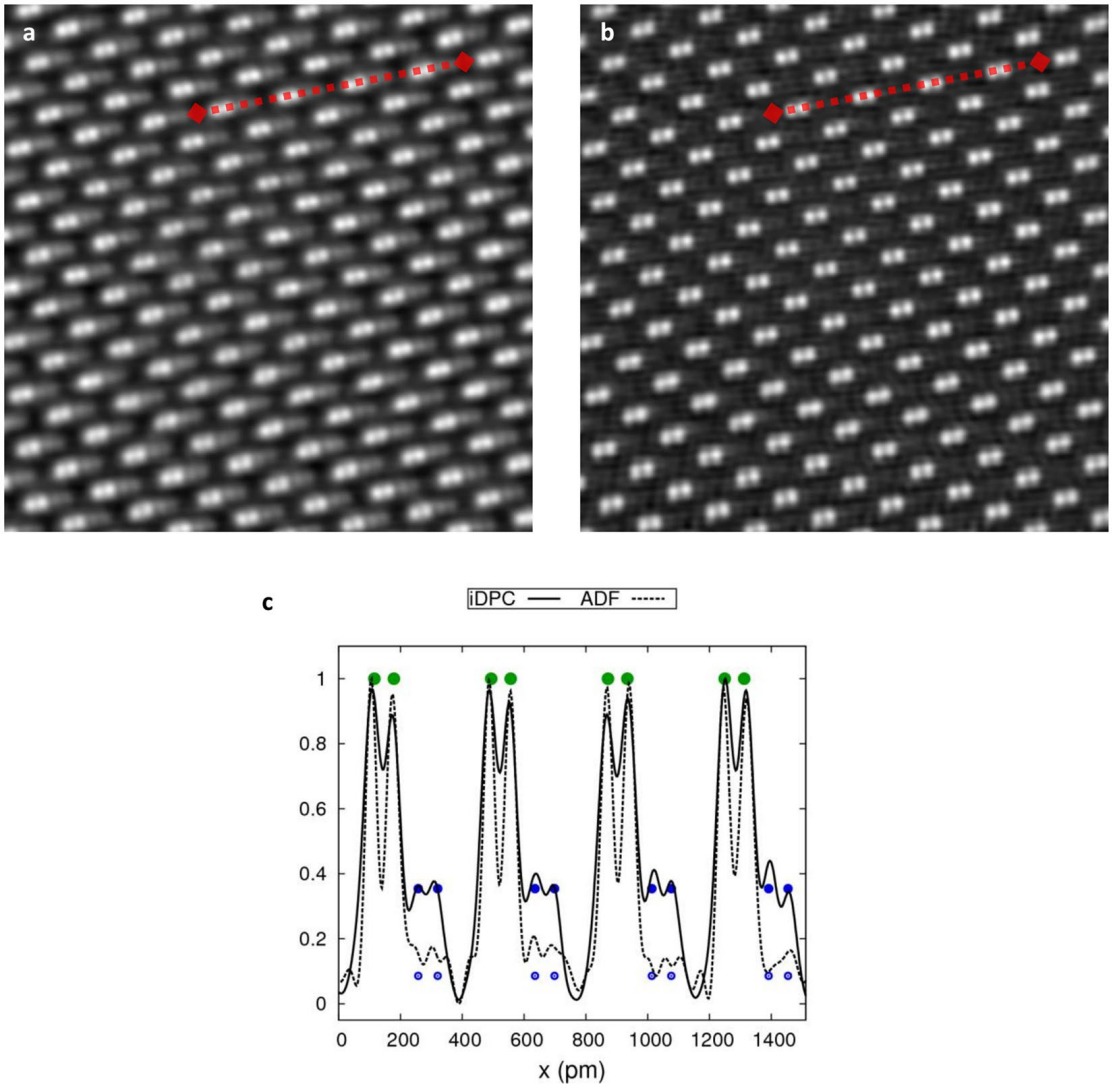
(**a**) iDPC- and (**b**) ADF-STEM images of GaN in [$10\bar{1}1$] orientation. Both images are CTF corrected using (1) and high pass filtered such that the large scale variatons are suppressed (same parameters as in Fig. 3). The FOV is 3.1 nm. (**c**) Normalized intensity profile plots of iDPC- (solid line) and ADF-STEM (dashed line) along the indicated red dashed lines. The green dots indicate the position and expected intensity (based on numerical simulations) of the Ga columns, the solid blue dots correspond to the N columns and expected intensity in the iDPC signal, the open blue dots correspond to the expected intensity of the ADF-STEM image.

Further quantitative analysis is possible if we analyze line profiles across the columns. Figure 4 shows the full (uncropped) iDPC-STEM image (Fig. 4a) of GaN in [$10\bar{1}1$] orientation corresponding to Fig. 3d together with the corresponding simultaneously recorded ADF-STEM image (Fig. 4b). Also shown are line profiles (Fig. 4c), taken at one specific location, and approximately 1.5 nm (4 unit repetitions) long, as indicated in the images. The iDPC profile clearly shows both the Ga and N dumbells which are nicely separated. The ADF signal shows only something of a shoulder with some wiggles, caused by the deconvolution on the much noisier raw ADF signal (line profiles of the raw images are included in the supplementary material). Further quantitative comparison of the line profiles in Fig. 4c shows that the contrast between Ga-Ga atom columns is roughly 2.5 times larger in the ADF- than in the iDPC-STEM image. This can easily be understood as the contrast in the ADF-STEM image is approximately the square of the contrast in a linear iDPC-STEM image resulting in narrower intensity peaks which have less overlap. The price to pay for this higher contrast, however, is the decrease in the sensitivity to the light elements in ADF-STEM image.

The crystallographic positions of Ga and N are also indicated in the line profile plots. The signal ratios between Ga and N atomic columns can be computed numerically from a STEM image simulation^7,34^. The peak intensities for a simulation containing single N and Ga atoms, using the atomic potentials from Kirkland^34^ yield a ratio of 0.35 for iDPC-STEM (solid blue dots in Fig. 4c) and 0.09 for ADF-STEM (open blue dots in Fig. 4c). As the GaN crystal in [$10\bar{1}1$] orientation has equal numbers of Ga an N in each column, the same ratios are also to be expected from the peak intensities for the crystal. The peak ratio obtained in Fig. 4c confirms this with high accuracy for the iDPC signal while it is slightly underestimated for the much noisier raw ADF signal.

**Figure 5:**
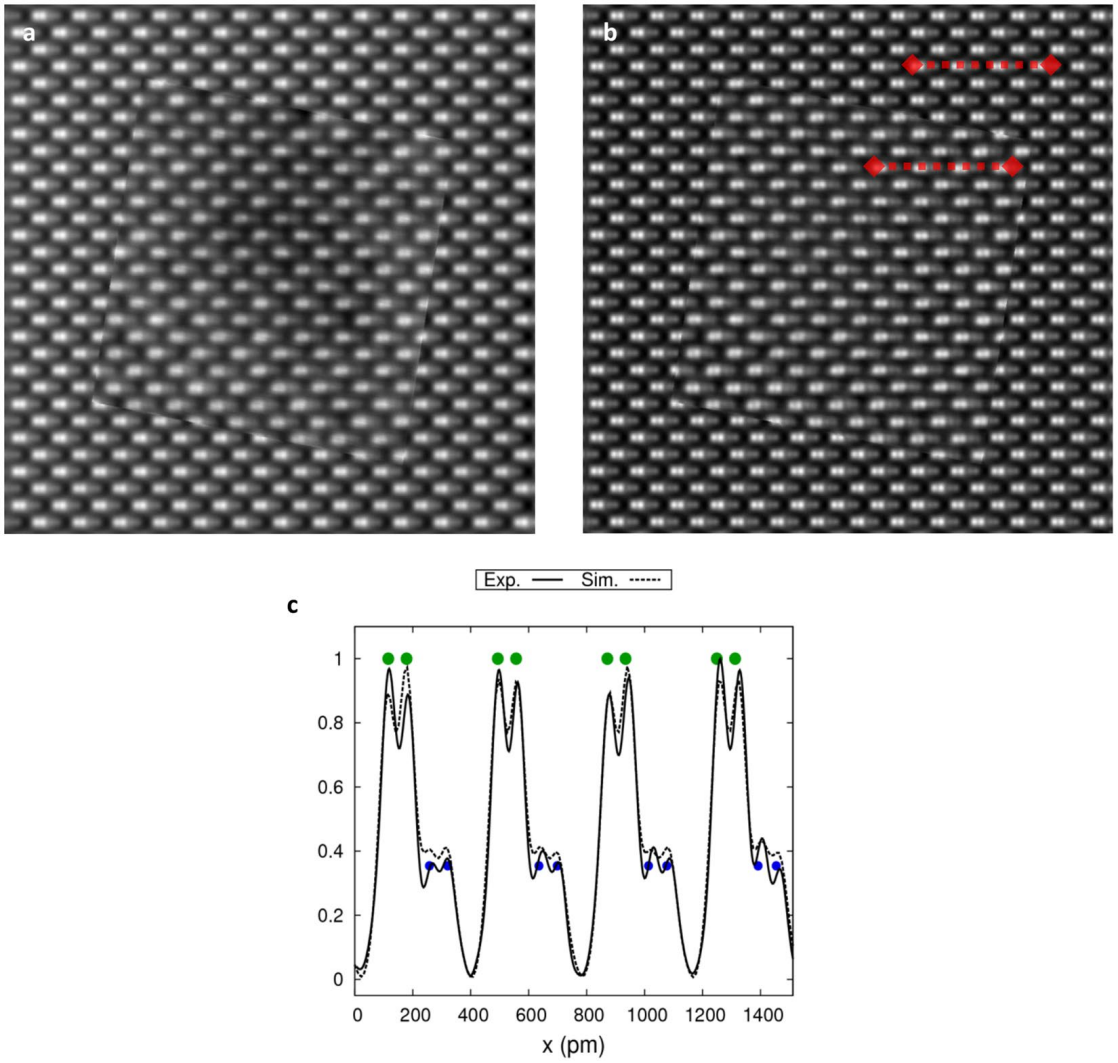
Simulated (background images) vs. experimental (inserted and rotated images) iDPC-STEM images of GaN in [$10\bar{1}1$] orientation: (**a**) raw and (**b**) CTF corrected and Gaussian high pass filtered as in Fig. 3. (**c**) Normalized intensity profiles (dashed line - simulation, solid line - experiment) along the red dashed lines indicated in (**b**). The FOV is 3.1 nm for the experimental and 5 nm for the simulated images. The thickness of the sample in the simulated image is 7.46 nm.

Figure 5 shows a comparison between the experimentally obtained iDPC-STEM image and a multi slice simulation using a GaN crystal sample in [$10\bar{1}1$] orientation with a thickness of 7.5 nm. Figure 5a shows the raw experimental image inserted in the simulated image, the experimental image is still recognizable as it contains low-frequency information not present in the simulation; Fig. 5b shows the same after CTF correction and filtering where the experimental image in the center is hardly distinguishable from the simulation surrounding it. The thickness of 7.5 nm was judged to give the best match from a set of simulations at different sample thicknesses, in the supplementary material results for two different thicknesses are included. The line profiles in Fig. 5c also confirm that the simulations and the experiment match closely.

## Discussion

We demonstrated the use of the direct phase imaging technique iDPC-STEM, its capability of imaging at a large field of view and showing light and heavy elements together, at the limit of contrast and resolution. The raw iDPC-STEM images of GaN as recorded without any post-processing reveal all relevant information, which is due to the possibility to focus and fine tune the probe on the fly during imaging. If desired the appearance of the relevant information, which, in this case reveals the crystal lattice of GaN in two different orientations ([$11\bar{2}0$] and [$10\bar{1}1$]) can be further perfected either during or after the acquisition, by applying standard image processing techniques like filtering and CTF correction. We demonstrated that resolution and contrast are also quantitatively accurate by obtaining the correct distances between the atoms and correct relative intensities of the atomic columns as predicted from simulations. As a reference, simultaneous ADF-STEM imaging was performed, where only heavy elements are revealed. We explained the results in both type of images by pointing out the appropriate formulation of the imaging formation process and indicating the objects and the CTFs explicitly.

Although in this work we concentrated on standard ADF- and iDPC-STEM imaging, all other DPC family images are simultaneously available. For completeness, the full “family” portrait is given in the supplementary material (Fig. S5).

## Methods

### Experimental

An aberration corrected FEI Themis 80–300 operated at 300 kV with convergence semi-angle of 29.4 mrad was used. With these settings the highest obtainable theoretical resolution is 33.5 pm (corresponding to $2{k}_{BF}$ in the Fourier domain where ${k}_{BF}$ is the frequency corresponding to the opening semi-angle of the beam. This does not include the effect of the finite source size). The probe corrector is able to correct aberrations up to 5^th^ order spherical aberration, C_5_. The following aberration coefficients were measured; A2 = 21 nm, B2 = 9 nm, C3 = −200 nm, A3 = 102 nm, S3 = 211 nm, A4 = 10.7 µm, D4 = 8.2 µm, B4 = 5.7 µm, C5 = 614 µm, A5 = 199 µm, S5 = 5 µm, and R5 = 21 µm (notation after Haider *et al*.^35^). Defocus, C1, and two-fold astigmatism, A1, were manually optimized. The illumination angle was chosen in order to balance the impact of the diffraction limit against residual coherent aberrations and, considering the defocus spread of 1.68 mm · eV, to minimize probe tails that would arise if a too large probe convergence angle is chosen.

In order to achieve the highest possible resolution with ADF-STEM, very thin samples are a necessity. To achieve this, a GaN sample was prepared and thinned/cleaned using a FEI Helios G4 FIB. The thickness at the positions where images were acquired was estimated to be less than 10 nm for the [$10\bar{1}1$] orientation and below 20 nm for the [$11\bar{2}0$] orientation. This was done by comparing experimental images to multi-slice simulations (included in the supplementary material). GaN images were recorded with pixel sizes around 3 pm. About 10 pA beam current was employed for STEM imaging to avoid beam damage and to take advantage of the high SNR ratio of iDPC-STEM imaging. The beam current is measured on the flu-screen which is carefully calibrated using a built-in Faraday cage.

### iDPC-STEM imaging

iDPC imaging is achieved using the 4-quadrant DF4 detector. In its simplest form, i.e. when the scanning direction is properly oriented w.r.t. the detector, the COM components are captured simply by the differences between signals of opposite quadrants (cf. supplementary Fig. S1). Analogous to the COM-STEM image a DPC-STEM vector image is obtained. It is proven^8,18^ that a scalar ${I}^{iDPC}({\boldsymbol{r}})$ image, analogues to the iCOM-STEM image ${I}^{iCOM}({\boldsymbol{r}})$, exists such that the DPC-STEM vector image is its gradient: ${{\boldsymbol{I}}}^{DPC}({\boldsymbol{r}})=\nabla {I}^{iDPC}({\boldsymbol{r}})$. This integrated image is obtained in the Fourier domain (with coordinate ${\boldsymbol{k}}$) using (cf. Eq. 20 in ref.^18^)

2 $${I}^{iDPC}({\boldsymbol{r}})={ {\mathcal F} }^{-1}\{\frac{{\boldsymbol{k}}\cdot  {\mathcal F} \{{{\boldsymbol{I}}}^{DPC}({\boldsymbol{r}})\}({\boldsymbol{k}})}{2\pi i{k}^{2}}\}({\boldsymbol{r}})$$

and directly represents the electrostatic potential of the sample $V({\boldsymbol{r}})$ if the sample is thin. Note that ${I}^{iDPC}({\boldsymbol{r}})$ cannot be determined at ${\boldsymbol{k}}=0$ from Eq. (2), which means that its DC component can be freely chosen. This is physically justified because $V({\boldsymbol{r}})$ can also be determined only up to a constant and its reference point (the DC component) can be chosen freely.

To be more precise, it was shown^8^ that the differential phase contrast (DPC) signal, formed by 4 segments (or any other number larger than 2), forms a vector image, ${{\boldsymbol{I}}}^{DPC}({\boldsymbol{r}})$, that can be integrated the same way as in Eq. (1) to obtain an iDPC scalar image, ${I}^{iDPC}({\boldsymbol{r}})$, which in the Fourier domain satisfies

3 $$ {\mathcal F} \{{I}^{iDPC}({\boldsymbol{r}})\}({\boldsymbol{k}})=CT{F}_{iS}({\boldsymbol{k}},\,{\boldsymbol{W}}({\boldsymbol{k}}\text{'}))\cdot  {\mathcal F} \{\sigma V({\boldsymbol{r}})\}({\boldsymbol{k}}).$$

Again, the ideal linear imaging form is obtained with the same phase object $\phi ({\boldsymbol{r}})=\sigma V({\boldsymbol{r}})$ as in the case of iCOM-STEM. This time the CTF becomes such that it includes the actual detector function ${\boldsymbol{W}}({\boldsymbol{k}})$.

Detailed expressions for $CT{F}_{iS}({\boldsymbol{k}},\,W({\boldsymbol{k}}\text{'}))$, as well as ${\boldsymbol{W}}({\boldsymbol{k}})$, are given in the supplementary material and, in much more depth, in section 4 of ref.^8^.

### ADF-STEM imaging

The image formation process of ADF-STEM^7,9,10,18^ results in the Fourier transform of the ADF-STEM image to be given as

4 $$ {\mathcal F} \{{I}^{ADF}({\boldsymbol{r}})\}({\boldsymbol{k}})=\,2\,C({\tilde{R}}_{N},W)\cdot \overline{ {\mathcal F} \{{|{\psi }_{in}({\boldsymbol{r}})|}^{2}\}}({\boldsymbol{k}})\cdot  {\mathcal F} \{1-\,\cos \,\phi ({\boldsymbol{r}})\}({\boldsymbol{k}})$$

where $C({\tilde{R}}_{N},W)$ is just a constant (i.e. independent of ${\boldsymbol{k}}$), a functional carrying a (weak) dependency on the sample and probe through a normalized autocorrelation function ${\tilde{R}}_{N}({\boldsymbol{r}})$ and the detector function $W({\boldsymbol{k}})$. For a given combination of sample and probe linear imaging is therefore obtained, with as object $1-\,\cos \,\phi ({\boldsymbol{r}})\approx {\phi }^{2}({\boldsymbol{r}})/2$ and a CTF given by $CT{F}_{ADF}({\boldsymbol{k}})=2\,C({\tilde{R}}_{N},W)\,\overline{ {\mathcal F} \{{|{\psi }_{in}({\boldsymbol{r}})|}^{2}\}}({\boldsymbol{k}})$. Details about $\,CT{F}_{ADF}({\boldsymbol{k}})$, the autocorrelation function $\,{\tilde{R}}_{N}({\boldsymbol{r}})$,$\,C({\tilde{R}}_{N},W)$, and $W({\boldsymbol{k}})$ are given in the supplementary material and, in much more depth, in section 3 of ref.^8^.

### Processing

iDPC-STEM images are obtained live with an optional high-pass filter applied to reduce the low frequency information in the image. CTF correction is done off-line and is described in more detail in the supplementary material.

### Simulation

Our multi-slice simulation closely follows the steps defined by Kirkland (Table 6.4 of ref.^34^) to obtain 4-quadrant detector images out of which DPC signals are formed as the difference of opposite quadrant images. The iDPC-STEM image is obtained in the same way as is done for the experiment images using (2). This is further illustrated and explained in Fig. S1 in the supplementary material. The parameters of the probe were set to match experimental values. The final iDPC-STEM images have been convolved with a Gaussian in order to include the effect of the finite source size.

## Electronic supplementary material

Supplementary Information
