# Supplementary material for: Phase contrast scanning transmission electron microscopy imaging of light and heavy atoms at the limit of contrast and resolution
Source: Sci Rep. 2018 Feb 8;8:2676. doi: 10.1038/s41598-018-20377-2 (PMC5805791; doi:10.1038/s41598-018-20377-2)
Supplement: Supplementary file 1 — Supplementary Information [file 41598_2018_20377_MOESM1_ESM.pdf]

Phase contrast scanning transmission electron microscopy imaging of light and heavy atoms at the limit of contrast and resolution

Emrah Yücelen, Ivan Lazić, Eric G.T. Bosch

Supplementary material

Contrast transfer function of iDPC-STEM: It was shown (ref. 8) that the differential phase contrast (DPC) signal, formed by 4 segments (or any other number larger than 2) which, in its simplest form, (i.e. when properly oriented w.r.t. the scanning direction, see Fig. S1) is just the difference between signals of opposite quadrants, forms a vector image, $\boldsymbol{I}^{DPC}(\boldsymbol{r})$, that can be integrated the same way as in Eq. (1) to obtain an iDPC scalar image, $I^{iDPC}(\boldsymbol{r})$, which in the Fourier domain ($\boldsymbol{k} = (k_x, k_y) = k_x\, \boldsymbol{e}_{k_x} + k_y\, \boldsymbol{e}_{k_y}$ and $k = |\boldsymbol{k}|$) satisfies

$$\mathcal{F}\{I^{iDPC}(\boldsymbol{r})\}(\boldsymbol{k}) = CTF_{iS}\big(\boldsymbol{k}, \boldsymbol{W}(\boldsymbol{k}')\big) \cdot \mathcal{F}\{\varphi(\boldsymbol{r})\}(\boldsymbol{k})\,. \tag{S1}$$

Here, the ideal linear imaging form is obtained with the object $\varphi(\boldsymbol{r}) = \sigma V(\boldsymbol{r})$ and the CTF given with

$$CTF_{iS}\big(\boldsymbol{k}, \boldsymbol{W}(\boldsymbol{k}')\big) = \frac{\boldsymbol{k} \cdot\ \boldsymbol{CTF}_S\big(\boldsymbol{k}, \boldsymbol{W}(\boldsymbol{k}')\big)}{2\pi i k^2}\,. \tag{S2}$$

This CTF is an integrated version of the DPC *vector* CTF, the $\boldsymbol{CTF}_S\big(\boldsymbol{k}, \boldsymbol{W}(\boldsymbol{k}')\big)$ given below, which includes the actual detector function $\boldsymbol{W}(\boldsymbol{k})$. In this work, the 4 quadrant detector shown in Fig. 1 is used $\boldsymbol{W}(\boldsymbol{k}) = W_x(\boldsymbol{k})\, \boldsymbol{e}_{k_x} + W_y(\boldsymbol{k})\, \boldsymbol{e}_{k_y}$, which with the appropriate scanning direction, becomes

$$\begin{aligned} W_x(\boldsymbol{k}) &= \frac{\pi}{2\sqrt{2}} k_{BF}\big(W_0(\boldsymbol{k}) - W_2(\boldsymbol{k})\big) \\ W_y(\boldsymbol{k}) &= \frac{\pi}{2\sqrt{2}} k_{BF}\big(W_1(\boldsymbol{k}) - W_3(\boldsymbol{k})\big) \end{aligned} \tag{S3}$$

where $W_j(\boldsymbol{k}) = \begin{cases} 1 & \frac{(2j-1)\pi}{4} < \vartheta < \frac{(2j+1)\pi}{4} \\ 0 & \text{elsewhere} \end{cases}$; $j \in \{0,1,2,3\}$ are detector functions of the four quadrants and k_{BF} is the frequency corresponding to the opening semi-angle of the beam (radius of the BF disk). To allow a general scanning direction w.r.t. the reference detector orientation described with (S3), this should be adjusted as explained in detail in ref. 8. Further, we have

$$\boldsymbol{CTF}_S\big(\boldsymbol{k}, \boldsymbol{W}(\boldsymbol{k}')\big) = CTF_S\big(\boldsymbol{k}, W_x(\boldsymbol{k}')\big)\boldsymbol{e}_{k_{px}} + CTF_S\left(\boldsymbol{k}, W_y(\boldsymbol{k}')\right) \boldsymbol{e}_{k_{py}} \tag{S4}$$

where

$$\begin{aligned} CTF_S\big(\boldsymbol{k}, W_x(\boldsymbol{k}')\big) = -i \cdot \Big(& \overline{\mathcal{F}\{\psi_{in}(\boldsymbol{r}) \cdot \mathcal{F}\{W_x(\boldsymbol{k}')\mathcal{F}^{-1}\{\overline{\psi_{in}(\boldsymbol{r}')}\}(\boldsymbol{k}')\}(\boldsymbol{r})\}(\boldsymbol{k})} + \\ & - \overline{\mathcal{F}\{\overline{\psi_{in}(\boldsymbol{r})} \cdot \mathcal{F}^{-1}\{W_x(\boldsymbol{k}')\mathcal{F}\{\psi_{in}(\boldsymbol{r}')\}(\boldsymbol{k}')\}(\boldsymbol{r})\}(\boldsymbol{k})}\Big). \end{aligned} \tag{S5}$$

and analogously for $CTF_S\left(\boldsymbol{k}, W_y(\boldsymbol{k}')\right)$. Here $\boldsymbol{k}'$ is a dummy variable of the inner Fourier transforms and $\bar{z}$ denotes the complex conjugate of z. Although, by looking at (S2) - (S5), the CTF of iDPC appears complicated it is actually not much different from the CTF of iCOM. It only reflects the symmetry and orientation of the detector w.r.t. the scanning direction as shown in Fig. S2 b and c.

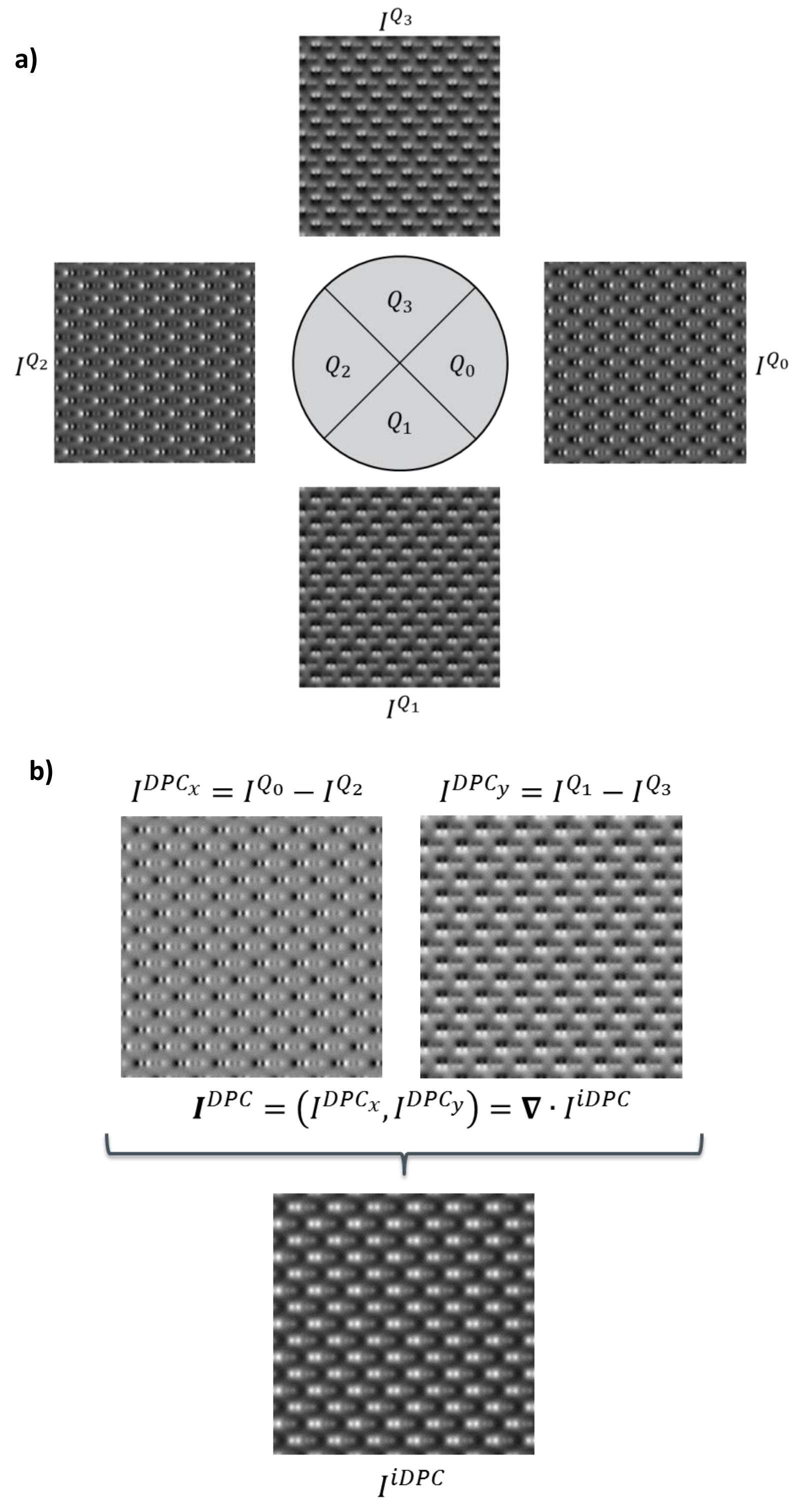

Figure S1: Overview of the steps in iDPC-STEM imaging using a 4 quadrant detector. a) Raw images from the four quadrants in the orientation shown in the center. b) DPCx and DPCy components (Cartesian coordinates) of the DPC-STEM vector image and the corresponding iDPC-STEM scalar image of which the DPC-STEM is the gradient. Scanning directions are from left to right and from top to bottom.

Contrast transfer function of ADF-STEM: It was shown in ref. 7 and discussed in detail in ref. 8 that the Fourier transform of the ADF-STEM image is given by

$$\mathcal{F}\{I^{ADF}(\boldsymbol{r})\}(\boldsymbol{k}) = CTF_{ADF}(\boldsymbol{k}) \cdot \mathcal{F}\{1-\cos\varphi(\boldsymbol{r})\}(\boldsymbol{k}) \tag{S6}$$

which, for a given sample and probe, has the form required for ideal linear imaging with as the object $1 - \cos\varphi(\boldsymbol{r}) \approx \varphi^2(\boldsymbol{r})/2$ and the CTF given by

$$CTF_{ADF}(\boldsymbol{k}) = 2\, C\big(\tilde{R}_N, W\big)\, \overline{\mathcal{F}\{|\psi_{in}(\boldsymbol{r})|^2\}(\boldsymbol{k})}\,. \tag{S7}$$

Here $C\big(\tilde{R}_N, W\big)$ is just a constant (i.e. independent of $\boldsymbol{k}$), obtained with the following functional

$$C\big(\tilde{R}_N, W\big) = \iint_{-\infty}^{\infty} \tilde{R}_N(\boldsymbol{r}')\, \mathcal{F}\{W(\boldsymbol{k})\}\,(\boldsymbol{r}')\, d^2\boldsymbol{r}' \tag{S8}$$

which carries the dependency of the sample and probe through $\tilde{R}_N(\boldsymbol{r}')$, the normalized autocorrelation function of the product $\big(1 - e^{i\varphi(\boldsymbol{r})}\big)\psi_{in}(\boldsymbol{r})$, were $\psi_{in}(\boldsymbol{r})$ is the probe. It can be approximated accurately with

$$\tilde{R}_N(r') = D_{\rho_1}(\mathrm{r}') \cdot \left(1 - \frac{\mathrm{r}'}{\rho_1}\right). \tag{S9}$$

Here, $D_{\rho_1}(r')$ is a unit disk $D_{\rho_1}(\mathrm{r}') \equiv \begin{cases} 1 & r' \le \rho_1 \\ 0 & r' > \rho_1 \end{cases}$ of radius ρ_1 (a parameter that depends on the sample and the beam opening semi-angle). Finally, the detector function $W(\boldsymbol{k})$ for ADF is defined as

$$W(\boldsymbol{k}) = \begin{cases} 1 & k_{min} \le |\boldsymbol{k}| \le k_{max} \\ 0 & \text{elsewhere} \end{cases} \tag{S10}$$

where $k_{min} > k_{BF}$ and k_{BF} is the bright field disk radius.

CTF correction: Using the known CTF of a linear imaging technique, the measured image, $I^{meas}(\boldsymbol{r})$, can be corrected to match the object more closely. The corrected image $I^{corr}(\boldsymbol{r})$ is then given by

$$I^{corr}(\boldsymbol{r}) = \mathcal{F}^{-1}\left\{\frac{\mathcal{F}\{I^{meas}(\boldsymbol{r})\}(\boldsymbol{k})}{CTF(\boldsymbol{k})}\right\}(\boldsymbol{r}) \tag{S11}$$

which is nothing else than a deconvolution operation.

Numerically (S11) has to be treated with care if the CTF comes close to zero. Therefore Wiener filtering is usually applied (see supplementary ref. 1). The Wiener filter takes the form

$$\Phi(\boldsymbol{k}) = \frac{|\mathcal{F}\{I^{meas}(\boldsymbol{r})\}(\boldsymbol{k})|^2 - |\mathcal{F}\{\eta(\boldsymbol{r})\}(\boldsymbol{k})|^2}{|\mathcal{F}\{I^{meas}(\boldsymbol{r})\}(\boldsymbol{k})|^2} \tag{S12}$$

where, $|\mathcal{F}\{\eta(\boldsymbol{r})\}(\boldsymbol{k})|^2$ is the estimated noise power spectrum. With this (S11) becomes

$$I^{corr}(\boldsymbol{r}) = \mathcal{F}^{-1}\left\{\mathcal{F}\{I^{meas}(\boldsymbol{r})\}(\boldsymbol{k}) \cdot \frac{\Phi(\boldsymbol{k})}{CTF(\boldsymbol{k})}\right\}(\boldsymbol{r}). \tag{S13}$$

As the CTF of any STEM technique is limited to $|\boldsymbol{k}| < 2k_{BF}$, the noise can be estimated from the power spectrum of the measured image outside of this circle in k-space. Due to the fact that the fast part of the scanning in the electron microscope is horizontal we construct a Wiener filter that is a function of the x-component of $\boldsymbol{k}$, by averaging the spectrum outside the circle containing the signal in the vertical direction.

Figure S2a illustrates how we deal with the part of the CTF curve (blue curve) that is close to zero (without actually being equal to zero). Instead of directly applying (S13) we truncate the inverse of the CTF (green curve representing 1/ $CTF(\boldsymbol{k})$) at a given maximum spatial frequency given by k_{LP}, where the index LP stands for Low-Pass. Furthermore, we follow the inverse of the CTF curve only up to a spatial frequency k_C, where the index C stands for correction. This results in the "practical" curve for the CTF correction that is applied to the measured data instead of 1/ $CTF(\boldsymbol{k})$ in (S13). The final corrected image will then have a CTF that is given by the black curve. The full 2D CTF of iCOM (as well as ADF) is shown in Fig. S2b. The corresponding CTF correction is rotationally symmetric and is shown in Fig. S2d.

Note that it is also possible to correct for the CTF of iDPC-STEM (equation (S2), Fig. S2c), instead of using the CTF of iCOM-STEM (equation (2)). The resulting correction is given in Fig. S2e. The effect on the image, however, is barely noticeable, indicating that the CTF of iDPC is indeed very close to the rotationally symmetric CTF of iCOM. Note also that the actual CTF of iDPC-STEM reflects the number, symmetry and orientation of segments used in the detection.

In Figure 3 in the main text, the actual parameters used were $k_C = 0.8\ k_{BF}$, and $k_{LP} = 1.0\ k_{BF}$ for the $[11\bar{2}0]$ orientation and $k_C = 1.3\ k_{BF}$, and $k_{LP} = 1.4\ k_{BF}$ for the $[10\bar{1}1]$ orientation. On top of this, we used a Gaussian high-pass filter that just removes the central part of the power spectrum.

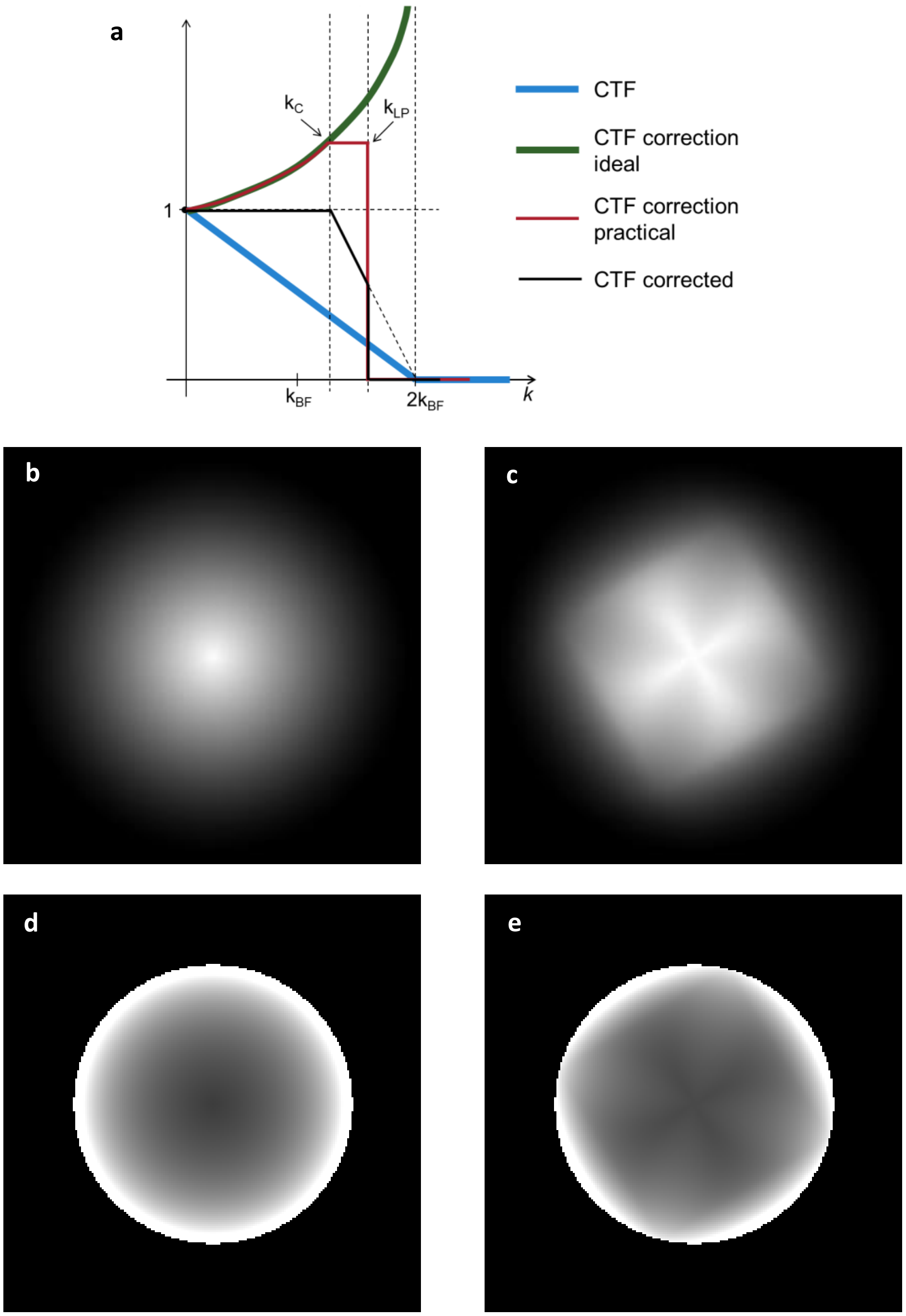

Figure S2: a) Illustration of the CTF correction; b) CTF of the iCOM-STEM for the optimized probe; c) CTF of iDPC-STEM for the optimized probe and detector oriented as in the experiment, 14 degrees w.r.t. the scanning direction from the situation given with (S3); d) resulting correction (corresponding to the red curve) using the CTF of iCOM and parameters $k_C = 1.3\, k_{BF}$, and $k_{LP} = 1.4\, k_{BF}$ as for GaN in $[10\bar{1}1]$ orientation; e) the same as in d) only using the CTF of iDPC with the 4 quadrant detector oriented as in the experiment.

We continue by showing the individual effects of applying just CTF correction and compared to just using filtering.

Figure S3 shows a comparison between raw iDPC- and ADF-STEM images together with their line profiles (left) and the corresponding CTF corrected images and line profiles (right). As already explained, the images on the right have a resulting corrected CTF given by the black curve in Fig. 1a. It is clear that this operation increases the sharpness of the image and helps with separation of the dumbbells. It is important that this operation completely preserves all low frequency information that was contained in the original raw images.

Figure S4 shows a comparison between band pass filtered (high pass Gaussian plus low pass filter) of the iDPC- and ADF-STEM images together with their line profiles (left) and the corresponding Gaussian high-pass filtered and CTF corrected images and line profiles (right). We see that just some simple filtering is capable of improving the visibility of the dumbbells and the clarity of the image, although not as clearly as pure CTF correction did in Fig. S3. Obviously the combination of filtering and CTF correction then gives the most optimal result. Note that this time low frequency information is removed.

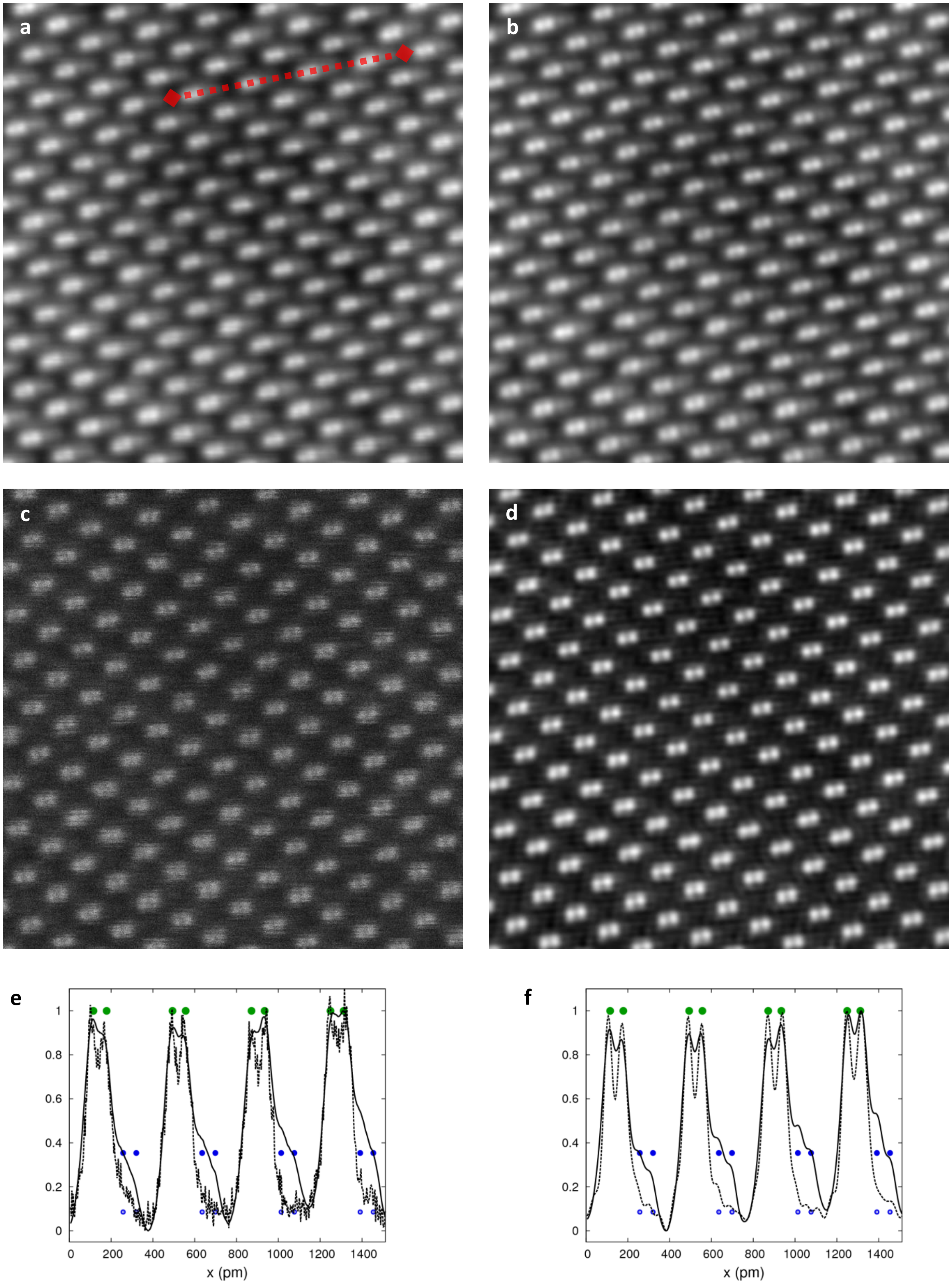

Figure S3: a),b) iDPC-STEM and c),d), ADF-STEM images with e),f) corresponding line profiles of GaN in $[10\bar{1}1]$ orientation along the line indicated in a). Raw images in a),c),e); CTF corrected images in b),d),f).

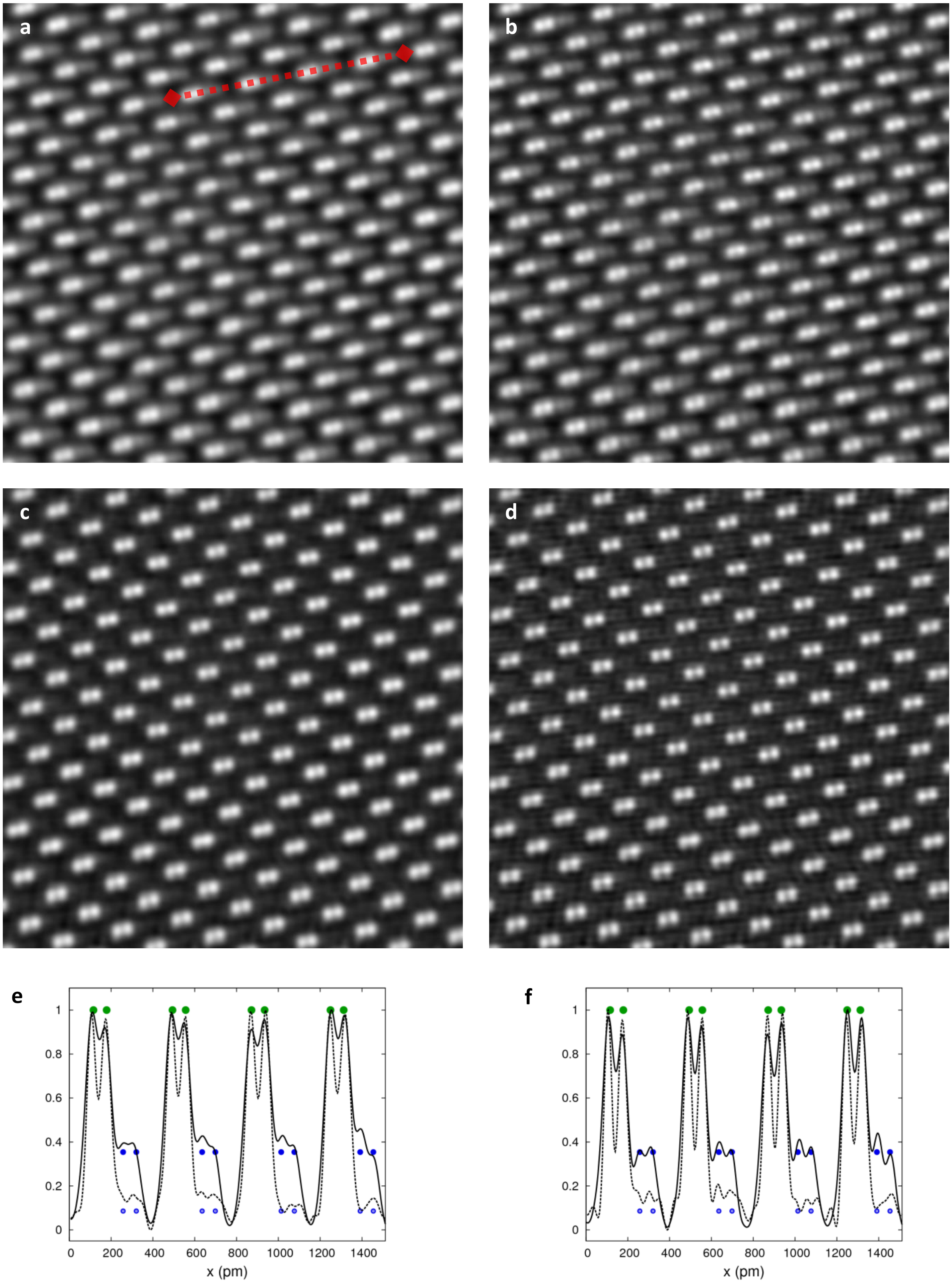

Figure S4: Analogous to Fig. S3. All images are high-pass filtered using a Gaussian blur with a width of $k_\sigma = 0.15\ k_{BF}$ and low pass filtered to the same value applied in the CTF correction $k_{LP} = 1.4\ k_{BF}$.

DPC "family portrait": In the main text we concentrated on the ADF- and iDPC-STEM images because they directly image the electrostatic potential (iDPC linearly and ADF quadratically) of a thin sample. The iDPC-STEM image is the only *scalar* STEM image that includes and also fully shows the full electrostatic potential information of the sample (including low and high frequency info and the contrast between light and heavy elements).

Although available during live acquisition as raw images, DPC and dDPC images can also easily be reproduced via the iDPC-STEM image. The DPC members of the family, consisting of the *vector* DPC-STEM image (which can be represented in Cartesian or spherical coordinates to give component images), is just the gradient of the iDPC-STEM image and represents the in-plane electric field of the thin sample. Similarly, the dDPC-STEM image is just the Laplacian of the iDPC-STEM image and represents the charge density of the thin sample. For completeness we show the full STEM imaging "album" for GaN $[10\bar{1}1]$ in orientation in Fig. S5.

Obtaining all DPC family images via the iDPC-STEM image by differentiation reduces the noise. This is due to a physical regularization which occurs due to the fact that integration is only allowed for conservative fields (i.e. the electric field), and therefore not for the noise[17] which will be reduced by integration.

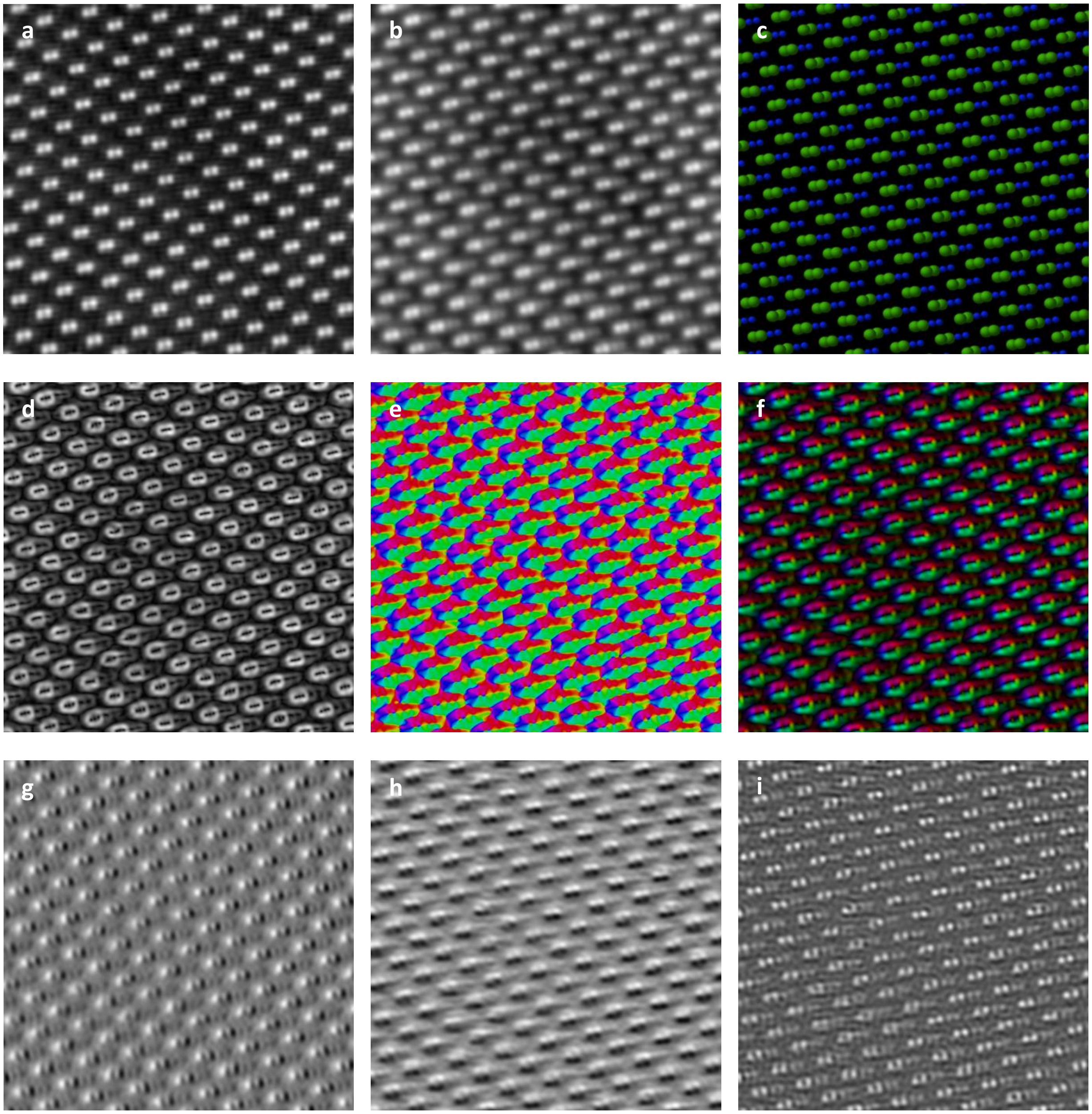

Figure S5: DPC “family portrait” for GaN in $[10\bar{1}1]$ orientation: a) ADF-STEM (square of the electrostatic potential of the sample), b) iDPC-STEM (electrostatic potential of the sample), c) crystal model, d) DPC-STEM intensity (electric field intensity), e) DPC-STEM direction (electric field direction), f) DPC-STEM (electric field vector using colorwheel for direction and color intensity for intensity) g) DPCx-STEM (inverted electric field x component), h) DPCy-STEM (inverted electric field y component), g) dDPC-STEM inverted (charge density). All DPC-STEM representations and dDPC-STEM were obtained via iDPC using noise regularization as shown in Fig. S6.

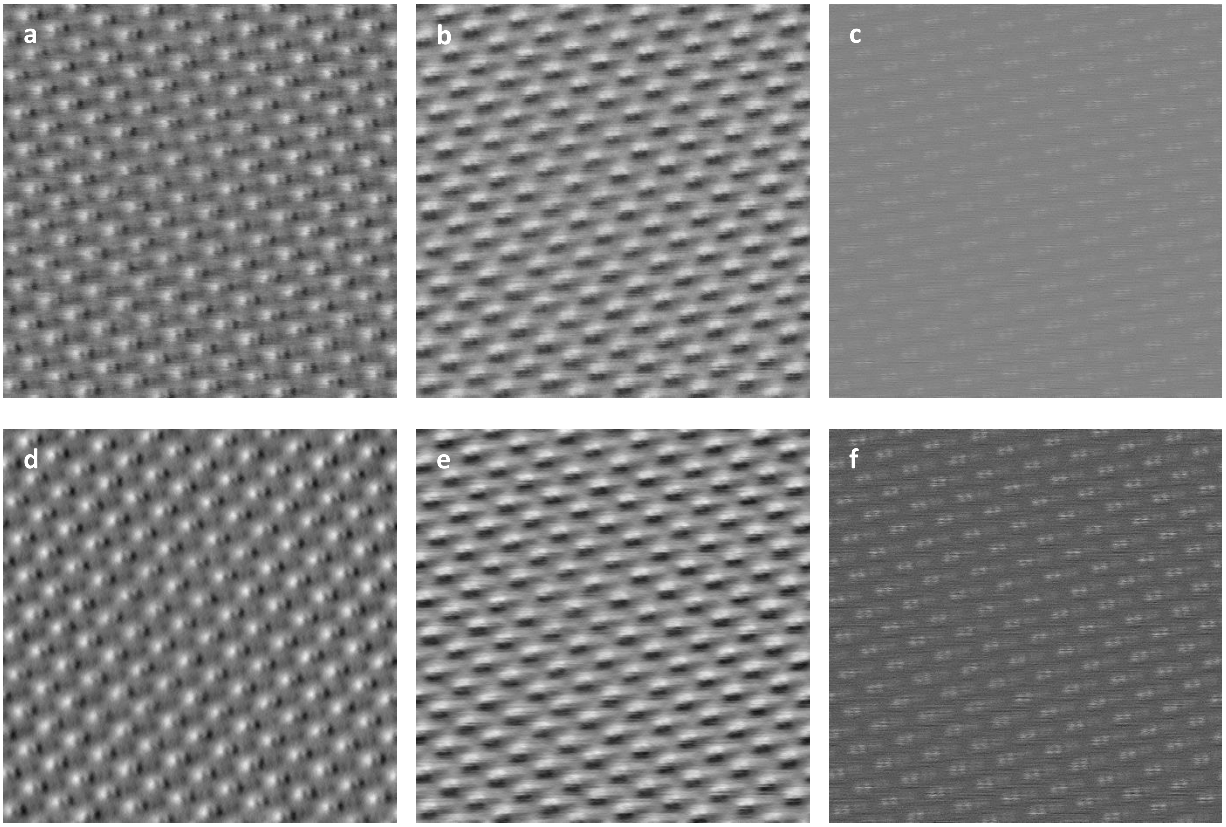

Figure S6. Top: a) raw DPCx-, b) raw DPCy- and c) raw inverted dDPC-STEM image of GaN in $[10\overline{1}1]$ orientation. Bottom: d) DPCx- via iDPC, e) DPCy- via iDPC and f) inverted dDPC-STEM via iDPC image of GaN in $[10\overline{1}1]$ orientation.

The difference between raw DPC-STEM images (shown in Cartesian coordinates) and inverted raw dDPC-STEM image w.r.t. the corresponding images obtained via iDPC-STEM are shown in Fig. S6.

Sample thickness: In the main text we considered the GaN sample under investigation to be thin. This allowed us to apply the theory valid for thin samples[8] to interpret the results. Here, we justify the assumption that a GaN sample of thickness below 10 nm can be considered thin. We also show that the thickness of our sample used in the experiment was certainly less than this.

The criterion that we apply for this purpose is a comparison with a multi-slice simulation of the sample for different thicknesses. In order for a sample to be considered as "thin", an iDPC-STEM image produced using a mono-slice simulation or equivalently using thin sample theory, should not differ significantly from the multi-slice image of the sample.

By comparing iDPC-STEM images for different sample thicknesses using the multi slice simulation, we notice that up to a certain thickness the simulated images look practically identical and then start differing significantly for larger thickness. This is shown in Fig. S7 where thickness 3.68 nm, 7.46 nm and 11.25 nm were used. While the images of for thicknesses of 3.68 nm and 7.46 nm look pretty much identical (and also very similar to a single-slice simulation), the image for 11.25 nm is significantly different. Placing the experimental image on top of the simulated ones, we see that that it matches the images of the two thinnest samples far better than the latter. The optimal match was obtained with the image of the 7.46 nm thick sample. With this result we can be quite confident that our sample was thinner than 10 nm and that images can be described using the thin sample theory.

The simulated images have a defocus of 2 nm into the sample and have been convolved with a Gaussian in order to include the effect of the source size. Due to the low frequency information in the raw experimental image it is easier to spot the experimental image inside the simulated image (left column in Fig. S7).

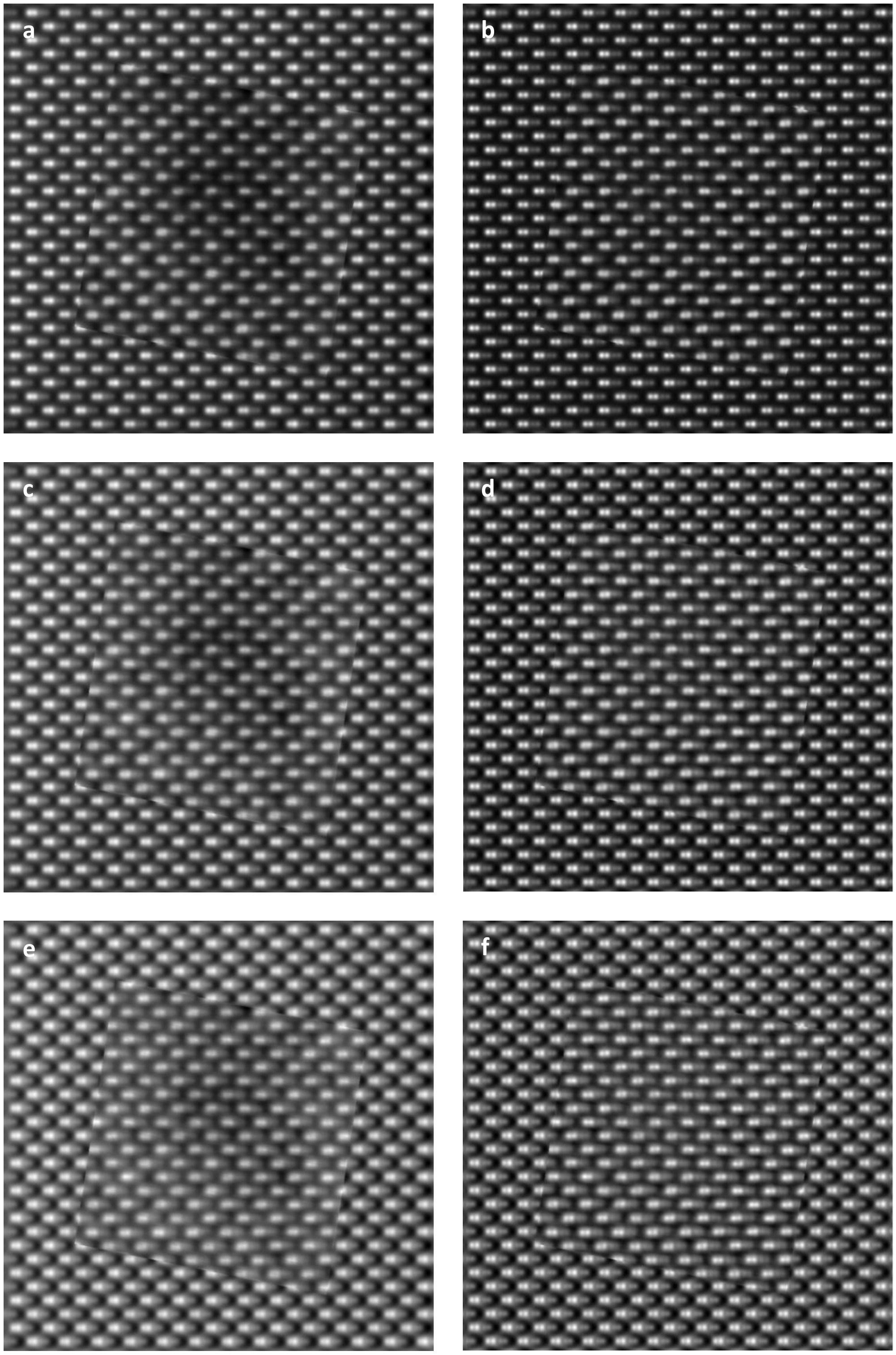

Figure S7: Comparison between experimental (central rotated square) and multi-slice simulation (background image) iDPC-STEM images. a,c,e) Raw and b,d,f) CTF corrected and Gaussian high pass filtered. Simulated thicknesses: a-b) 3.68 nm; c-d) 7.46 nm and e-f) 11.25 nm.

Supplementary References

1. Press, W.H., Teukolsky, S.A., Vetterling, W.T. and Flannery, B.P., 1996. Numerical recipes in C (Vol. 2). Cambridge: Cambridge university press.
